# Supplementary material for: Research on China’s embodied carbon import and export trade from the perspective of value-added trade
Source: PLoS One. 2021 Nov 18;16(11):e0258902. doi: 10.1371/journal.pone.0258902 (PMC8601465; doi:10.1371/journal.pone.0258902)
Supplement: S1 Appendix — (DOCX) [file pone.0258902.s001.docx]

Annexed table 1. Names of 26 industries

| S1 Agriculture | S14 Construction |
| --- | --- |
| S2 Fishing | S15 Maintenance and Repair |
| S3 Mining and Quarrying | S16 Wholesale Trade |
| S4 Food & Beverages | S17 Retail Trade |
| S5 Textiles and Wearing Apparel | S18 Hotels and Restraurants |
| S6 Wood and Paper | S19 Transport |
| S7 Petroleum, Chemical and Non-Metallic Mineral Products | S20 Post and Telecommunications |
| S8 Metal Products | S21Finacial Intermediation and Business Activities |
| S9 Electrical and Machinery | S22 Public Administration |
| S10 Transport Equipment | S23 Education, Health and Other Services |
| S11 Other Manufacturing | S24 Private Households |
| S12 Recycling | S25 Others |
| S13 Electricity, Gas and Water | S26 Re-export & Re-import |
