# Supplementary material for: Research on China’s embodied carbon import and export trade from the perspective of value-added trade
Source: PLoS One. 2021 Nov 18;16(11):e0258902. doi: 10.1371/journal.pone.0258902 (PMC8601465; doi:10.1371/journal.pone.0258902)
Supplement: S2 Appendix — (DOCX) [file pone.0258902.s002.docx]

Annexed table 2.Names of 190countries（regions）

| Afghanistan | Benin | Chile | Estonia | Hungary | Lesotho | Morocco | Peru | Slovakia | Tunisia |
| --- | --- | --- | --- | --- | --- | --- | --- | --- | --- |
| Albania | Bermuda | China | Ethiopia | Iceland | Liberia | Mozambique | Philippines | Slovenia | Turkey |
| Algeria | Bhutan | Colombia | Fiji | India | Libya | Myanmar | Poland | Somalia | Turkmenistan |
| Andorra | Bolivia | Congo | Finland | Indonesia | Liechtenstein | Namibia | Portugal | South Africa | Former USSR |
| Angola | Bosnia and Herzegovina | Costa Rica | France | Iran | Lithuania | Nepal | Qatar | South Sudan | Uganda |
| Antigua | Botswana | Croatia | French Polynesia | Iraq | Luxembourg | Netherlands | South Korea | Spain | Ukraine |
| Argentina | Brazil | Cuba | Gabon | Ireland | Macao SAR | Netherlands Antilles | Moldova | Sri Lanka | UAE |
| Armenia | British Virgin Islands | Cyprus | Gambia | Israel | Madagascar | New Caledonia | Romania | Sudan | UK |
| Aruba | Brunei | Czech Republic | Georgia | Italy | Malawi | New Zealand | Russia | Suriname | Tanzania |
| Australia | Bulgaria | Cote dIvoire | Germany | Jamaica | Malaysia | Nicaragua | Rwanda | Swaziland | USA |
| Austria | Burkina Faso | North Korea | Ghana | Japan | Maldives | Niger | Samoa | Sweden | Uruguay |
| Azerbaijan | Burundi | DR Congo | Greece | Jordan | Mali | Nigeria | San Marino | Switzerland | Uzbekistan |
| Bahamas | Cambodia | Denmark | Greenland | Kazakhstan | Malta | Norway | Sao Tome and Principe | Syria | Vanuatu |
| Bahrain | Cameroon | Djibouti | Guatemala | Kenya | Mauritania | Gaza Strip | Saudi Arabia | Taiwan | Venezuela |
| Bangladesh | Canada | Dominican Republic | Guinea | Kuwait | Mauritius | Oman | Senegal | Tajikistan | Viet Nam |
| Barbados | Cape Verde | Ecuador | Guyana | Kyrgyzstan | Mexico | Pakistan | Serbia | Thailand | Yemen |
| Belarus | Cayman Islands | Egypt | Haiti | Laos | Monaco | Panama | Seychelles | TFYR Macedonia | Zambia |
| Belgium | Central African Republic | El Salvador | Honduras | Latvia | Mongolia | Papua New Guinea | Sierra Leone | Togo | Zimbabwe |
| Belize | Chad | Eritrea | Hong Kong | Lebanon | Montenegro | Paraguay | Singapore | Trinidad and Tobago | ROW |
