# Supplementary material for: Research on China’s embodied carbon import and export trade from the perspective of value-added trade
Source: PLoS One. 2021 Nov 18;16(11):e0258902. doi: 10.1371/journal.pone.0258902 (PMC8601465; doi:10.1371/journal.pone.0258902)
Supplement: Attachment — Submitted filename: Response to editor and reviewers.docx [file pone.0258902.s003.docx]

**Response to editor and reviewers**

First of all, we would like to thank the editors and reviewers for pointing out the shortcomings of this paper. We have revised the paper according to the review comments, and the corrections are marked in red. The following is our reply to the comments of the editors and reviewers:

Journal Requirements:

When submitting your revision, we need you to address these additional requirements.

1. Please ensure that your manuscript meets PLOS ONE's style requirements, including those for file naming. The PLOS ONE style templates can be found at

https://journals.plos.org/plosone/s/file?id=wjVg/PLOSOne_formatting_sample_main_body.pdf and

<https://journals.plos.org/plosone/s/file?id=ba62/PLOSOne_formatting_sample_title_authors_affiliations.pdf>

Re: The manuscript has been modified in accordance with the PLOS ONE format.

2. Thank you for stating the following financial disclosure:

This work was supported by the Natural Science Foundation of China under Grant [number 71704070]; Natual Social Science Fund(17BJY061); Outstanding Youth Fund of Gansu Province [number 20JR5RA206]; Gansu Provincial Higher Education Research Project [number 2020A-058]; and Program of Lanzhou University of Finance and Economics under Grant [number Lzufe2018B-06].

Please state what role the funders took in the study. If the funders had no role, please state: "The funders had no role in study design, data collection and analysis, decision to publish, or preparation of the manuscript."

If this statement is not correct you must amend it as needed.

Please include this amended Role of Funder statement in your cover letter; we will change the online submission form on your behalf.

Re: Guangyao Deng was awarded four grants from Natural Science Foundation of China under Grant [number 71704070]; Outstanding Youth Fund of Gansu Province [number 20JR5RA206]; Gansu Provincial Higher Education Research Project [number 2020A-058]; and Program of Lanzhou University of Finance and Economics under Grant [number Lzufe2018B-06]. Xiaofang Yue was awarded grant from Social Science Fundation of China [number 17BJY061]. The specific roles of these authors are articulated in the author contributions’ section. The funders had no other role in study design, data collection and analysis, decision to publish, or preparation of the manuscript.

Additional Editor Comments:

In addition to comments from the reviewers, I have several comments for authors to consider.

1) it would enhance the paper by discussing and comparing their results with other relevant studies on this topic together/after presenting their results.

Re: the revised draft has added a discussion section, please refer to the discussion section in the revised draft for details.

2) What are the limitations and potential problems of the study, such as strict assumptions of the input-output methods, data quality, and the role of price changes over time?

Reply: The revised draft added some limitations of the research method. The input-output model has the following three assumptions: (1) homogeneity assumption: Assuming that each product department produces only one homogeneous product, and the products of one product department cannot be replaced by the other. That is, the consumption structure, production process and economic use are the same, which is also called the aggregate of homogeneous products. (2) The proportionality hypothesis: The output of the department is directly proportional to the input. Only in this way can output and input be guaranteed to be linear functions. (3) Assumption of relative stability of consumption coefficients: it is assumed that in a certain period (1 year), all kinds of consumption coefficients are relatively stable. This paper is based on the above three assumptions.

In addition, the data required for compiling the global input-output table are collected from different countries, so the data in the input-output table are the data after integration, and there will be some differences between the integrated data and the original data.

The changes of price are eliminated by the deflator, which is described in the data sources section.

3) Any potential directions for future research?

Re: The revised draft adds the further research direction of the article. The further research direction of this paper is to use the complex network analysis method to study the network characteristics of the embodied carbon trade in major countries around the world, and use the regression model to study the influencing factors of the embodied carbon trade.

4) As the analysis is based on data until 2015, will it still be valid or to what extent will it be valid for the recent years, particularly after the COVID-2019 pandemic?

Re: This problem is explained at the end of the paper, as follows:

Due to the availability of data, the research deadline of this paper is only 2015. In recent years, especially since the outbreak of COVID-19, China's foreign trade situation may have undergone major changes, and thus China's embodied carbon trade will also undergo major changes. Due to the impact of the epidemic, China's foreign trade declined sharply from January 2020 to May 2020. As the Chinese government adopted a series of extraordinary policies to stabilize foreign trade, China's foreign trade gradually recovered from June 2020. Overall, the foreign trade in 2020 still increased slightly compared with that in 2019. In this way, the amount of embodied carbon trade may increase slightly, and the main destination of embodied carbon export and the main source of embodied carbon import may change, so the amount of embodied carbon trade in various industries will also be different from the results in 2015.

5) English needs to be improved.

Re: We have carefully edited English language.

Reviewers' comments:

Reviewer #1: Exploring trade from the value-added perspective is a very important topic worth digging into. The starting point of this paper is new and insightful. The authors have done solid work by using input-output analysis and structural decomposition analysis. Some suggestions on polishing the paper into better shape are as follows:

Re: Thanks.

The focus of the paper, especially the result section, should be put on the importance of incorporating value-added perspective and the difference it made on the results, i.e., how does traditional statistics distort facts and over-estimate trade imbalance and how does incorporating value-added perspective change traditional accounting. Results Section One (Accounting results of embodied carbon trade) largely deals with conclusions already extensively presented. This section is suggested to be shortened.

Re: As suggested by the reviewer, the calculation results of China's embodied carbon trade have been mentioned in many articles. However, different from the existing literature, the calculation in this part is carried out from the perspective of value-added trade. In addition, there is a one-to-one correspondence between the results and structural decomposition analysis in Figure 1-5 and the results in Table 1-5 (the structural decomposition analysis is based on the difference between 2015 and 2006 data in Figure 1-5)., so it is not appropriate to delete any of them.

In addition, according to the suggestions of reviewers, in order to reduce the length of this part, only the results are listed, and the explanation of the results is put in the discussion section of the paper.

The writing of the introduction section is subpar compared with the rest of the paper. A detailed list of previous studies on embodied carbon emissions using input-output analysis is redundant. The introduction of the input-output analysis seems a bit unprofessional. For a better introduction, the authors are suggested to refer to the following literature.

Wu X D , Guo J L , Han M Y , et al. An overview of arable land use for the world economy: From source to sink via the global supply chain[J]. Land Use Policy, 2018, 76:201-214.

Chen G Q , Wu X D , Guo J L , et al. Global overview for energy use of the world economy: Household-consumption-based accounting based on the world input-output database (WIOD)[J]. Energy Economics, 2019, 81.

Re: In the literature review of the revised manuscript, two literatures proposed by the reviewer are added, as follows:

Researchers usually extend the conventional input-output model to the environmental input-output model to study the embodied carbon trade [5], virtual land [6] and embodied energy[7].

Due to the differences in the process of using input-output models to calculate the embodied carbon trade in different literatures, the revised version deleted the following:

Generally, the direct carbon emission coefficient is calculated by combining the total input and carbon emissions of each sector, and then, the embodied carbon import and export trade volume is calculated by using the Leontief inverse matrix and the trade volume of some finally used products in the input–output table.

Some minor comments:

This sentence in the abstract is too colloquial: “countries that have a lot of trade with China”.

Re：“countries that have a lot of trade with China” is revised as “countries trading with China”

The first sentence in the introduction in grammatically incorrect. “It” cannot be used when not immediately followed by a referent.

Re：The first sentence in the introduction is revised as “With rapid economic growth, China’s consumption of various types of fossil energy has been increasing.”

The usage of CO2 and carbon dioxide should be consistent in the paper.

Re: The revised draft has changed carbon dioxide into CO2 (except for references ).

The language in the introduction section is suggested to be thoroughly polished by a native.

Re: The language has been polished by a native.

This paper is suggested for major revision.

Re: Thank you for the opportunity to revise this paper.

Reviewer #2: This article measures the embodied carbon emissions between China and other major countries or regions from the perspective of the value-added chain and uses factor decomposition methods to analyze the calculation results. Although there are many studies on embodied carbon emissions from the perspective of the value-added chain, this article focuses on the analysis of the carbon emissions correlation between China and other regions, which also has certain research significance. The research method of the whole article is appropriate, and the logical structure is clear. It is recommended to publish it after minor revisions. Before proceeding further, I have some small suggestions:

Re: Thanks.

1. The literature review should be expanded. Such as Hongguang Liu et al. studied the carbon emissions embodied in value added chains in China (Journal of Cleaner Production, 2015,103: 362-370). Su and Ang (2017; Energy Economics 65, 137-147) firstly propose the aggregate embodied intensity (AEI) framework by defining the AEI indicator as the ratio of embodied energy (or emissions) to embodied value added using the I-O framework. Recently, the AEI analysis has been further extended to the transmission layer by Su et al. (2019; Energy Economics 83, 345-360). The AEI indicator at the higher level can be represented as a weighted sum of the AEI indicators at the lower level. There are already studies using the AEI indicators at the country level, such as China (Su and Ang, 2017; Su et al., 2019) and India (Zhu et al., 2018; Applied Energy 230, 1545-1556), and at the global level, such as Yang and Su (2019; Applied Energy 253, 113552) and Duan and Yan (2019; Energy Economics 83, 540-554) using the WIOD database. These studies also use the SDA technique to investigate the driving forces to the changes observed.

Re: The revised draft supplements the above literature in the part of literature review and also mentions the above literature in the newly added discussion.

2. Some detailed description of the data source should be given, such as the industry and region division information.

Re：The specific names of the 26 industries in the 190 countries (regions) can be found in the EORA database (<http://www.worldmrio.com/>).The revised draft supplemented industry and country (region) information in the form of appendix. Details are as follows:

Annexed table 1. Names of 26 industries

| S1 Agriculture | S14 Construction |
| --- | --- |
| S2 Fishing | S15 Maintenance and Repair |
| S3 Mining and Quarrying | S16 Wholesale Trade |
| S4 Food & Beverages | S17 Retail Trade |
| S5 Textiles and Wearing Apparel | S18 Hotels and Restraurants |
| S6 Wood and Paper | S19 Transport |
| S7 Petroleum, Chemical and Non-Metallic Mineral Products | S20 Post and Telecommunications |
| S8 Metal Products | S21Finacial Intermediation and Business Activities |
| S9 Electrical and Machinery | S22 Public Administration |
| S10 Transport Equipment | S23 Education, Health and Other Services |
| S11 Other Manufacturing | S24 Private Households |
| S12 Recycling | S25 Others |
| S13 Electricity, Gas and Water | S26 Re-export & Re-import |

Annexed table 2.Names of 190countries（regions）

| Afghanistan | Benin | Chile | Estonia | Hungary | Lesotho | Morocco | Peru | Slovakia | Tunisia |
| --- | --- | --- | --- | --- | --- | --- | --- | --- | --- |
| Albania | Bermuda | China | Ethiopia | Iceland | Liberia | Mozambique | Philippines | Slovenia | Turkey |
| Algeria | Bhutan | Colombia | Fiji | India | Libya | Myanmar | Poland | Somalia | Turkmenistan |
| Andorra | Bolivia | Congo | Finland | Indonesia | Liechtenstein | Namibia | Portugal | South Africa | Former USSR |
| Angola | Bosnia and Herzegovina | Costa Rica | France | Iran | Lithuania | Nepal | Qatar | South Sudan | Uganda |
| Antigua | Botswana | Croatia | French Polynesia | Iraq | Luxembourg | Netherlands | South Korea | Spain | Ukraine |
| Argentina | Brazil | Cuba | Gabon | Ireland | Macao SAR | Netherlands Antilles | Moldova | Sri Lanka | UAE |
| Armenia | British Virgin Islands | Cyprus | Gambia | Israel | Madagascar | New Caledonia | Romania | Sudan | UK |
| Aruba | Brunei | Czech Republic | Georgia | Italy | Malawi | New Zealand | Russia | Suriname | Tanzania |
| Australia | Bulgaria | Cote dIvoire | Germany | Jamaica | Malaysia | Nicaragua | Rwanda | Swaziland | USA |
| Austria | Burkina Faso | North Korea | Ghana | Japan | Maldives | Niger | Samoa | Sweden | Uruguay |
| Azerbaijan | Burundi | DR Congo | Greece | Jordan | Mali | Nigeria | San Marino | Switzerland | Uzbekistan |
| Bahamas | Cambodia | Denmark | Greenland | Kazakhstan | Malta | Norway | Sao Tome and Principe | Syria | Vanuatu |
| Bahrain | Cameroon | Djibouti | Guatemala | Kenya | Mauritania | Gaza Strip | Saudi Arabia | Taiwan | Venezuela |
| Bangladesh | Canada | Dominican Republic | Guinea | Kuwait | Mauritius | Oman | Senegal | Tajikistan | Viet Nam |
| Barbados | Cape Verde | Ecuador | Guyana | Kyrgyzstan | Mexico | Pakistan | Serbia | Thailand | Yemen |
| Belarus | Cayman Islands | Egypt | Haiti | Laos | Monaco | Panama | Seychelles | TFYR Macedonia | Zambia |
| Belgium | Central African Republic | El Salvador | Honduras | Latvia | Mongolia | Papua New Guinea | Sierra Leone | Togo | Zimbabwe |
| Belize | Chad | Eritrea | Hong Kong | Lebanon | Montenegro | Paraguay | Singapore | Trinidad and Tobago | ROW |

2. It is best to add a discussion section to discuss the comparison between the results of this article and similar research results.

Reply: The discussion is added in the revised draft.
